# Calcium Silicate-Based Cements Associated with Micro- and Nanoparticle Radiopacifiers: Physicochemical Properties and Bioactivity

**DOI:** 10.1155/2015/874283

**Published:** 2015-02-23

**Authors:** Roberta Bosso-Martelo, Juliane Maria Guerreiro-Tanomaru, Raqueli Viapiana, Fábio Luis Camargo Vilella Berbert, Maria Inês Basso Bernardi, Mario Tanomaru-Filho

**Affiliations:** ^1^Department of Restorative Dentistry, Araraquara Dental School, University of Estadual Paulista (UNESP), 14801-903 Araraquara, SP, Brazil; ^2^Institute of Physics, University of São Paulo (USP), São Carlos, SP, Brazil

## Abstract

*Objective*. The aim of this study was to evaluate the physicochemical properties and bioactivity of two formulations of calcium silicate-based cements containing additives (CSCM) or resin (CSCR), associated with radiopacifying agents zirconium oxide (ZrO_2_) and niobium oxide (Nb_2_O_5_) as micro- and nanoparticles; calcium tungstate (CaWO_4_); and bismuth oxide (Bi_2_O_3_). MTA Angelus was used as control. *Methods*. Surface features and bioactivity were evaluated by scanning electron microscopy and the chemical composition by energy dispersive X-ray spectrometry (EDS-X). *Results*. CSCM and CSCR presented larger particle sizes than MTA. Hydroxyapatite deposits were found on the surface of some materials, especially when associated with the radiopacifier with ZrO_2_ nanoparticles. All the cements presented calcium, silicon, and aluminum in their composition. *Conclusion*. Both calcium silicate-based cements presented composition and bioactivity similar to MTA when associated with the radiopacifiers evaluated.

## 1. Introduction

Mineral trioxide aggregate (MTA) has been classified as calcium silicate-based cement [1] and its extensive clinical indication is attributed to its biocompatibility, alkalinity (pH value 12.5), sealing ability [2], and bioactivity [3, 4]. MTA has Portland cement as the main component in its composition and bismuth oxide (Bi_2_O_3_) to promote radiopacity [5, 6]. The physical, chemical, mechanical, biologic, and antimicrobial properties of calcium silicate-based cements and MTA are similar [7–11].

Calcium silicate-based cements, which have greater consistency and are easier to manipulate, are evaluated as an alternative to MTA. For example, Biodentine (Septodont, Saint-Maur-des-Fossés, France) corresponds to a tricalcium silicate-based cement [12, 13], which has been developed with indications similar to those of MTA, and as a dentine substitute [14].

The Bi_2_O_3_ present in MTA may compromise the physical, mechanical, and biologic properties of the cement [5, 6, 15]. Therefore, new radiopacifiers have been evaluated as alternatives to Bi_2_O_3_. For example, zirconium oxide (ZrO_2_) and calcium tungstate o (CaWO_4_) when incorporated into Portland cement result in cements with radiopacity exceeding the minimum value recommended by the ANSI/ADA Specification 57 [16]. Furthermore, both ZrO_2_ micro- and nanoparticles have demonstrated bioactive potential [17–19]. The association of ZrO_2_ and CaWO_4_ with Portland cement showed no cytotoxicity [9]. Niobium oxide (Nb_2_O_5_) may promote radiopacity and improve the biologic properties of materials due to its biocompatibility [20] and bioactivity [21]. Its use in the form of nanoparticles has shown bioactive and antimicrobial potential [22].

Bioactivity is a desirable property for retrofilling cement, because a bioactive material has the capacity to develop a stable bond with live tissue by means of hydroxyapatite deposition [23]. The association of calcium silicate-based cement with different radiopacifying agents such as ZrO_2_, Nb_2_O_5_, or CaWO_4_ with different particle size (nano- or microparticles) may favor the bioactive potential of materials. Therefore, the aim of this study was to analyze the surface, chemical composition, and bioactivity of two calcium silicate-based cements with different chemical compositions in association with different radiopacifiers.

## 2. Material and Methods

### 2.1. Sample

Two calcium silicate-based cements with different chemical compositions were evaluated, associated with micro- and nanoparticles of radiopacifying agents (Table 1). The nanoparticles of radiopacifiers were obtained by the polymeric precursor method at the Physics Institute of São Carlos (University of São Paulo, São Carlos, Brazil). The polymeric precursor method is based on metal citrate polymerization with ethylene glycol. A hydroxycarboxylic acid such as citric acid is commonly used as a chelating agent for the cations in an aqueous solution. The addition of a polyalcohol such as ethylene glycol leads to the formation of an organic ester. The polymerization is promoted by heating at around 120°C resulting in a homogeneous resin in which the cations are distributed evenly throughout the organic matrix. The resin is then calcined to produce the desired oxides.

The ZrO_2_ supports were prepared by the polymeric precursor method, from the precursor salt ZrO(NO_3_)_2_·*x*H_2_O (Alfa Aesar). Aqueous solutions of this salt were prepared, mixed, and added to an aqueous solution of citric acid (held at 60°C), with constant stirring. Subsequently, ethylene glycol (HOCH2CH2OH) was added to polymerize the citrate by a polyesterification reaction (at 120°C). The citric acid :  metal molar ratio was 3 : 1, while the citric acid  : ethylene glycol mass ratio was 60 : 40. The resulting polymer resin was then calcined at 300°C for 4 h, and after 600°C/2 h to produce ZrO_2_ crystalline particles.

An aqueous solution of niobium ammonium oxalate {NH_4_[NbO(C_2_O_4_)_2_(H_2_O)](H_2_O)N(CBMM)} was prepared and ammonium hydroxide was dropped upon thereafter. The niobium hydroxide precipitated was filtered and washed to eliminate oxalate ions and dissolved into a citric acid (CA) aqueous solution ([CA]/[Nb] = 3) and filtered. The niobium content in the solution was precisely determined by gravimetric analysis. The solution was stirred for 2 h at 70°C to promote the complex reaction. Ethylene glycol (EG) was added to the mixture with mass ratio 60 : 40. The translucent solution was heated and stirred over several hours. A polymerization process started during the water evaporation, resulting in a highly viscous solution. This resin was heated in an electric furnace at 300°C for 4 h. The resulting black and soft mass was milled and calcined in an electric furnace for 2 h over alumina slabs at 700°C/2 h.

The size of particles obtained for ZrO_2_ was 74 nm and for Nb_2_O_5_ it was 83 nm, which were confirmed by the Brunauer-Emmett-Teller surface area analysis and confirmed with scanning electron microscope. The materials were manipulated with distilled water in accordance with the proportions shown in Table 1.

### 2.2. Surface and Chemical Composition Analyses

For analysis of the surface morphology of the different experimental groups, the materials were manipulated and inserted into cylindrical molds 6 mm in diameter and 12 mm high. The specimens were kept in an oven at 37°C and immersed in distilled water for 28 days. After this period, the test specimens were dried with absorbent paper and kept in a desiccator containing silica, under vacuum, for 24 hours. The specimens were embedded in resin and polished in an automatic polishing machine (EcoMet 250 Grinder-Polisher Family, Illinois, USA). After being dried again, the specimens were placed on stubs, bathed in carbon, and examined by scanning electron microscopy (JEOL JSM 6610LV, Tokyo, Japan) at different magnifications (50x, 500x, and 1000x) in secondary backscattered electron mode. All the analyses were performed at 18 kV and SS 68. Furthermore, energy dispersive X-ray spectrometry (EDS-X) (Thermo Scientific, Madison, USA) analysis was performed for the images obtained at 1000x magnification.

### 2.3. Bioactivity

All cements were manipulated, compacted into cylindrical moulds measuring 1 mm high × 7.5 mm in diameter. After the materials were set in an incubator at 37°C and 100% humidity, samples were immersed in a standard phosphate buffered saline solution at 37°C for 30 days. Samples were placed on silica gel and soda lime and placed in an incubator for 12 hours to dry. Then, they were carbon coated for electrical conductivity. Surface microstructural assessment of the cements was performed under the scanning electron microscope (SEM) in secondary electron mode. Energy dispersive spectroscopy (EDS) was also performed after and before soaking in a standard phosphate buffered saline.

## 3. Results

### 3.1. Surface and Chemical Composition Analyses

**Figure 1:**
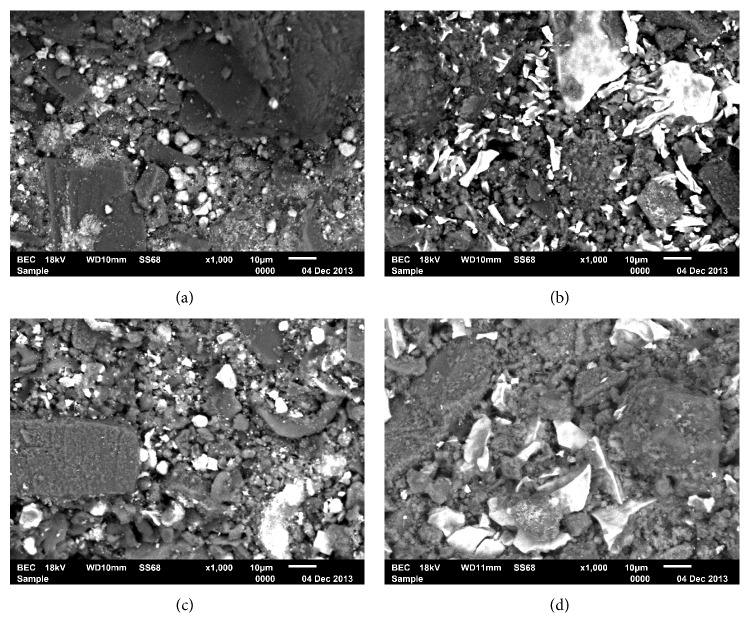
Backscattered electron micrography (1000x magnification) of CSCM samples associated with radiopacifiers: (a) ZrO_2_ micro, (b) ZrO_2_ nano, (c) Nb_2_O_5_ micro, and (d) Nb_2_O_5_ nano.

**Figure 2:**
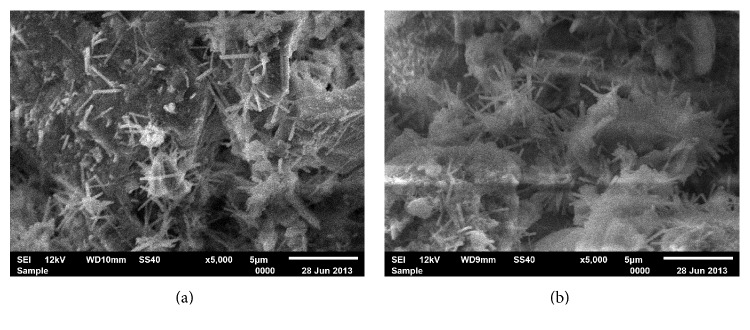
Scanning electron micrography by secondary electrons after hydration of cements, with honey-comb aspect of calcium silicate hydrate and needle-shaped ettringite crystals ((a) and (b)).

Electronic micrographs for the calcium silicate-based cements were represented in Figure 1. MTA was used as control. By EDS-X analysis, all the materials demonstrated peaks of calcium, silicone, and aluminum, indicating an aluminate phase that is characteristic of Portland-type cements and differently from pure tricalcium silicate-based cements. The EDS analysis of the CSCM and CSCR before and after soaking in a standard phosphate buffered saline is shown in Table 2. The CSCM and CSCR particle sizes were larger than those of MTA. All the different radiopacifiers used were visible in the electronic micrographs. The radiopacifiers had a brilliant appearance due to their high atomic mass. The nanoparticulate ZrO_2_ presented particles with larger sizes than those of microparticulate ZrO_2_. Cement hydration was evident from the presence of calcium silicate hydrate and ettringite in the secondary electron images at higher magnifications (Figure 2).

### 3.2. Bioactivity

**Figure 3:**
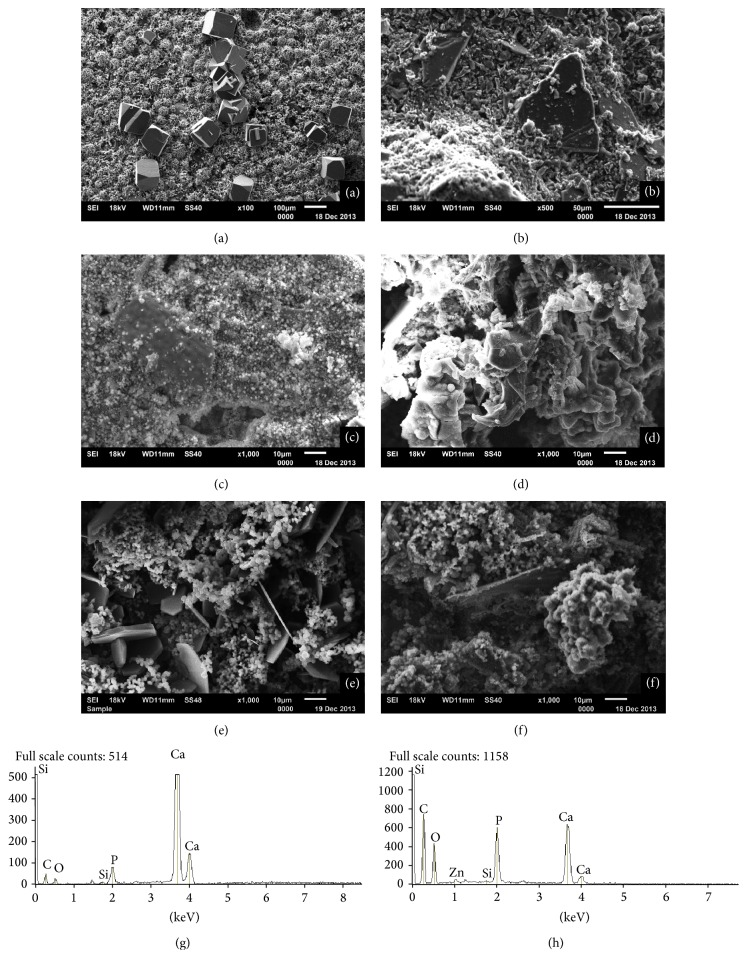
Secondary electron micrographs of (a) CSCM + ZrO_2_ nano, (b) CSCR + ZrO_2_ nano, (c) CSCM + Nb_2_O_5_ micro, (d) CSCR + Nb_2_O_5_ nano, (e) CSCM, and (f) CSCR, and energy dispersive X-ray spectrometry analysis of (g) CSCM and (h) CSCR after the materials were immersed in a standard phosphate buffered saline solution for 30 days.

The micrographs of samples after the bioactivity assay, in images by secondary electron scanning of the materials, are represented in Figure 3. All the cements presented a similar microstructure. The surface of materials presented a granular appearance, covered with small particles rich in calcium and phosphorous as indicated by the EDS analysis (Figure 3). Hexagon and cubic crystals measuring around 10–40 micrometers in size were visible on the surface of some of the materials, particularly for the cements evaluated with the association of nanoparticulate ZrO_2_ as indicated in the EDS analyses; these crystals are rich in calcium and oxygen (Figure 3).

## 4. Discussion

An ideal retrofilling material should promote sealing, present low solubility, be biocompatible, and demonstrate bioactive potential. Calcium silicate-based cements have good interaction with bone forming cells [24], and their bioactive potential [3] is responsible for the clinical success when these cements are used.

The replacement of Bi_2_O_3_ by ZrO_2_ associated with Portland cement demonstrated adequate physical and mechanical properties and bioactivity [25, 26]. Another possibility of the use of ZrO_2_ is using it in its nanoparticulate form, because it demonstrated biocompatibility and cytocompatibility [27] and improved the mechanical and physical properties of the materials [28, 29].

Metals such as niobium have deserved their outstanding place for use in dental materials, because of presenting excellent resistance to corrosion, not being allergenic or toxic [30], and being biocompatible [20], in addition to showing the capacity to promote apatite formation [21]. In the nanoparticulate form Nb_2_O_5_ presents antimicrobial activity [22]. CaWO_4_ has been studied as a radiopacifying agent associated with Portland cement [9, 31] presenting adequate biocompatibility [9], in addition to not altering the mechanical property and final setting time of Portland cement [31].

Analysis of the size and shape of MTA particles showed that this cement presents a homogeneous surface and small sized particles [32]. Materials with smooth and regular surfaces may promote less tissue irritation [33]. Dammaschke et al. [34] affirmed that the surface characteristics of a material may indicate its biocompatibility, as it has a direct influence on cell adhesion and distribution. According to Ha et al. [35] cements with smaller particles have greater disposition to absorb humidity. Salem Milani et al. [36] observed that when MTA cement comes into contact with body fluid, it presents hexagonal crystals with well-defined edges, amorphous crystals, and some of the needle type, unequally distributed throughout the entire material surface.

The cements evaluated and MTA cement are calcium silicate-based materials. All are derived from Portland cement; however, they present some differences in their composition and are manipulated with distilled water. The two calcium silicate-based cements used in this study had additives, pigments, and aggregates in their compositions, and the CSCR had a resin component. Scanning electron microscopy demonstrated that the CSCMs and CSCRs presented a surface morphology typical of Portland cement-based materials with particles of different sizes, while MTA had smaller and more homogeneous particles, according to Dammaschke et al. [34].

The radiopacifiers studied demonstrated different particle sizes and morphologies. The cements with particulate ZrO_2_ formed agglomerates with distinct morphologies, while the cements to which nanoparticulate Nb_2_O_5_ was added presented smaller and more dispersed particles. The secondary electron micrography demonstrated the process of cement hydration, with the formation of calcium silicate hydrate and ettringite. EDS-X analysis of the MTA cements and two calcium silicate-based cements with different chemical compositions indicated that all the materials presented similarity in their components, such as the elements calcium, aluminum, and silicone. Asgary et al. [37] observed that Gray MTA presents crystals approximately 8 times larger than those of White MTA, which reveals that White MTA presents a mixture with a finer texture than Gray MTA. Furthermore, both cements present calcium, silicone, and bismuth in their composition.

According to the literature, the bioactivity of MTA has been attributed to its capacity of hydroxyapatite production, when it is in the presence of a phosphate solution [38]. Since the Ca^2+^ and OH^−^ resulting from the dissociation of calcium hydroxide react with the phosphorous ions in the solution, this results in hydroxyapatite crystals on the surface of the material [39]. Therefore, the precipitation of hydroxyapatite* in vitro* on the surface of a material when it is in contact with a phosphate solution indicates its bioactivity [40]. In this study was observed the bioactivity of the materials, regardless of the addition or not of the radiopacifier (Figure 3). Generally speaking, the cements presented a similar microstructure. Hexagonal or cubic crystals were also observed, which corresponded to the hydroxyapatite formed. The EDS analysis showed that these crystals were rich in calcium and oxygen and presented phosphate peak after hydration in standard phosphate buffered saline (Figure 3) that suggests an apatite formation.

The concept of bioactivity is intimately correlated with biointeractivity, that is, the capacity to exchange information within a biologic system [41]. This means that a bioactive material reacts chemically with the fluids of the body in a manner compatible with the tissue repair processes [42]. Formosa et al. [43], by means of scanning electron microscopy, dispersive energy X-ray, X-ray diffraction, and optical profilometry characterization techniques, observed that tricalcium silicate is more bioactive than Portland cement.

Greater presence of hydroxyapatite crystals was observed when the cements were associated with nanoparticulate ZrO_2_. The high level of calcium and phosphorous deposition when the calcium silicate-based cements are associated with the ZrO_2_ nanoparticles indicates the formation of a layer of hydroxyapatite, thus reinforcing its bioactive potential. The greater degree of bioactivity of ZrO_2_ may be explained by the rapid dissolution of Ca^2+^ ions when in a phosphate solution and by the rapid nucleation of the Ca^2+^ and P^5+^ ions on the surface of the powder [18]. Previous studies [44, 45] have pointed out that nanoparticles present a higher degree of bioactivity than microparticles, which is in agreement with our findings.

## 5. Conclusions

Considering the results obtained in this study, all the associations presented a composition similar to that of MTA and presented bioactivity. Therefore, it was concluded that the calcium silicate-based cements evaluated presented the potential for use as an alternative to MTA when associated with the radiopacifiers studied.

## Figures and Tables

**Table 1:** Group of materials evaluated and powder-liquid ratio.

Material	Powder-liquid ratio
White^*^ MTA	1 g-300 *µ*L
Calcium silicate-based cement containing additives (CSCM)^**^	1 g-360 *µ*L
CSCM + 30% zirconium oxide (Zr_2_O) microparticles^****^	1 g-200 *µ*L
CSCM + 30% zirconium oxide (Zr_2_O) nanoparticles^*****^	1 g-360 *µ*L
CSCM + 30% niobium oxide (Nb_2_O_5_) microparticles^****^	1 g-340 *µ*L
CSCM + 30% niobium oxide (Nb_2_O_5_) nanoparticles^*****^	1 g-390 *µ*L
CSCM + 20% bismuth (Bi_2_O_3_) microparticles^****^	1 g-260 *µ*L
CSCM + 30% calcium tungstate (CaWO_4_) microparticles^******^	1 g-200 *µ*L
Calcium silicate resin-based cement (CSCR)^***^	1 g-360 *µ*L
CSCR + 30% zirconium oxide (Zr_2_O) microparticles^****^	1 g-235 *µ*L
CSCR + 30% zirconium oxide (Zr_2_O) nanoparticles	1 g-340 *µ*L
CSCR + 30% niobium oxide (Nb_2_O_5_) microparticles	1 g-380 *µ*L
CSCR + 30% niobium oxide (Nb_2_O_5_) nanoparticles	1 g-380 *µ*L
CSCR + 20% bismuth (Bi_2_O_3_) microparticles	1 g-250 *µ*L
CSCR + 30% calcium tungstate (CaWO_4_) microparticles	1 g-220 *µ*L

^*^MTA, Angelus, Londrina, Brazil.

^**^Usina Fortaleza ICMF Ltda., Barueri, SP, Brazil (composition: mineral aggregates, additives, and pigments).

^***^Ligatex Ind. e Com. Ltda., Rio Claro, SP, Brazil (composition: mineral aggregates, additives, resins, and pigments).

^****^Sigma-Aldrich Brasil Ltda., São Paulo, SP, Brazil.

^*****^Laboratório de Nanotecnologia, Instituto de Física de São Carlos, SP, Brazil.

^******^Sigma-Aldrich, St. Louis, MO, USA.

**Table 2:** EDS analysis of CSCM and CSCR before and after soaking in standard phosphate buffered saline (PBS).

	CSCM		CSCR	
	Before soaking in PBS	After soaking in PBS	Before soaking in PBS	After soaking in PBS
Chemical components	C, O, Ca, Si, Al, Mg and Br	C, O, Ca, Si and P	C, O, Ca, Si and Al	C, O, Ca, Si and P

## References

[1] Darvell B. W., Wu R. C. T. (2011). MTA—an hydraulic silicate cement: review update and setting reaction. *Dental Materials*.

[2] Adel M., Nima M. M., Kojoori S. S., Oliaie H. N., Naghavi N., Asgary S. (2012). Comparison of endodontic biomaterials as apical barriers in simulated open apices. *ISRN Dentistry*.

[3] Camilleri J., Sorrentino F., Damidot D. (2013). Investigation of the hydration and bioactivity of radiopacified tricalcium silicate cement, Biodentine and MTA Angelus. *Dental Materials*.

[4] Parirokh M., Torabinejad M. (2010). Mineral trioxide aggregate: a comprehensive literature review-part III: clinical applications, drawbacks, and mechanism of action. *Journal of Endodontics*.

[5] Camilleri J. (2011). Evaluation of the effect of intrinsic material properties and ambient conditions on the dimensional stability of white mineral trioxide aggregate and Portland cement. *Journal of Endodontics*.

[6] Coomaraswamy K. S., Lumley P. J., Hofmann M. P. (2007). Effect of bismuth oxide radiopacifier content on the material properties of an endodontic Portland cement-based (MTAlike) system. *Journal of Endodontics*.

[7] Islam I., Kheng Chng H., Jin Yap A. U. (2006). Comparison of the physical and mechanical properties of MTA and portland cement. *Journal of Endodontics*.

[8] Chang S. W., Shon W. J., Lee W., Kum K. Y., Baek S. H., Bae K. S. (2010). Analysis of heavy metal contents in gray and white MTA and 2 kinds of Portland cement: a preliminary study. *Oral Surgery, Oral Medicine, Oral Pathology, Oral Radiology and Endodontology*.

[9] Gomes Cornélio A. L., Salles L. P., da Paz M. C., Cirelli J. A., Guerreiro-Tanomaru J. M., Filho M. T. (2011). Cytotoxicity of Portland cement with different radiopacifying agents: a cell death study. *Journal of Endodontics*.

[10] Guerreiro-Tanomaru J. M., Gomes-Cornélio A. L., Andolfatto C., Salles L. P., Tanomaru-Filho M. (2012). pH and antimicrobial activity of portland cement associated with different radiopacifying agents. *ISRN Dentistry*.

[11] de Souza E. T. G., Nunes Tameirão M. D., Roter J. M., de Assis J. T., de Almeida Neves A., de-Deus G. A. (2013). Tridimensional quantitative porosity characterization of three set calcium silicate-based repair cements for endodontic use. *Microscopy Research and Technique*.

[12] Laurent P., Camps J., de Méo M., Déjou J., About I. (2008). Induction of specific cell responses to a Ca_3_SiO_5_-based posterior restorative material. *Dental Materials*.

[13] Laurent P., Camps J., About I. (2012). Biodentine(TM) induces TGF-beta1 release from human pulp cells and early dental pulp mineralization. *International Endodontic Journal*.

[14] Pérard M., le Clerc J., Meary F., Pérez F., Tricot-Doleux S., Pellen-Mussi P. (2013). Spheroid model study comparing the biocompatibility of Biodentine and MTA. *Journal of Materials Science: Materials in Medicine*.

[15] Camilleri J., Montesin F. E., Papaioannou S., McDonald F., Pitt Ford T. R. (2004). Biocompatibility of two commercial forms of mineral trioxide aggregate. *International Endodontic Journal*.

[16] ANSI/ADA (2000). *Specification 57: Endodontic Sealing Materia*.

[17] Dehestani M., Ilver L., Adolfsson E. (2012). Enhancing the bioactivity of zirconia and zirconia composites by surface modification. *Journal of Biomedical Materials Research Part B: Applied Biomaterials*.

[18] Karunakaran G., Suriyaprabha R., Manivasakan P., Yuvakkumar R., Rajendran V., Kannan N. (2013). Screening of in vitro cytotoxicity, antioxidant potential and bioactivity of nano- and micro-ZrO_2_ and -TiO_2_ particles. *Ecotoxicology and Environmental Safety*.

[19] Sarkar D., Swain S. K., Adhikari S., Reddy B. S., Maiti H. S. (2013). Synthesis, mechanical properties and bioactivity of nanostructured zirconia. *Materials Science and Engineering C*.

[20] Denry I. L., Holloway J. A., Nakkula R. J., Walters J. D. (2005). Effect of niobium content on the microstructure and thermal properties of fluorapatite glass-ceramics. *Journal of Biomedical Materials Research Part B: Applied Biomaterials*.

[21] Karlinsey R. L., Yi K., Duhn C. W. (2006). Nucleation and growth of apatite by a self-assembled polycrystalline bioceramic. *Bioinspiration and Biomimetics*.

[22] Wang Z., Lee Y. H., Wu B. (2010). Anti-microbial activities of aerosolized transition metal oxide nanoparticles. *Chemosphere*.

[23] Czarnecka B., Coleman N. J., Shaw H., Nicholson J. W. (2008). The use of mineral trioxide aggregate in endodontics—status report. *Dental and Medical Problems*.

[24] Pelliccioni G. A., Ciapetti G., Cenni E. (2004). Evaluation of osteoblast-like cell response to Proroot MTA (mineral trioxide aggregate) cement. *Journal of Materials Science: Materials in Medicine*.

[25] Camilleri J., Cutajar A., Mallia B. (2011). Hydration characteristics of zirconium oxide replaced Portland cement for use as a root-end filling material. *Dental Materials*.

[26] Cutajar A., Mallia B., Abela S., Camilleri J. (2011). Replacement of radiopacifier in mineral trioxide aggregate; characterization and determination of physical properties. *Dental Materials*.

[27] Liu X., Huang A., Ding C., Chu P. K. (2006). Bioactivity and cytocompatibility of zirconia (ZrO_2_) films fabricated by cathodic arc deposition. *Biomaterials*.

[28] Gillani R., Ercan B., Qiao A., Webster T. J. (2010). Nanofunctionalized zirconia and barium sulfate particles as bone cement additives. *International Journal of Nanomedicine*.

[29] Rodrigues D. C., Gilbert J. L., Hasenwinkel J. M. (2010). Two-solution bone cements with cross-linked micro and nano-particles for vertebral fracture applications: effects of zirconium dioxide content on the material and setting properties. *Journal of Biomedical Materials Research Part B: Applied Biomaterials*.

[30] Gladwin M., Bagby M. (2004). *Clinical Aspect of Dental Materials: Theory, Practice, and Cases*.

[31] Tanomaru-Filho M., Morales V., da Silva G. F. (2012). Compressive strength and setting time of MTA and portland cement associated with different radiopacifying agents. *ISRN Dentstry*.

[32] Komabayashi T., Spångberg L. S. W. (2008). Comparative analysis of the particle size and shape of commercially available mineral trioxide aggregates and Portland cement: a study with a flow particle image analyzer. *Journal of Endodontics*.

[33] Anthonappa R. P., King N. M., Martens L. C. (2013). Is there sufficient evidence to support the long-term efficacy of mineral trioxide aggregate (MTA) for endodontic therapy in primary teeth?. *International Endodontic Journal*.

[34] Dammaschke T., Gerth H. U. V., Züchner H., Schäfer E. (2005). Chemical and physical surface and bulk material characterization of white ProRoot MTA and two Portland cements. *Dental Materials*.

[35] Ha W. N., Kahler B., Walsh L. J. (2014). Particle size changes in unsealed mineral trioxide aggregate powder. *Journal of Endodontics*.

[36] Salem Milani A., Rahimi S., Froughreyhani M., Vahid Pakdel M. (2013). Effect of blood contamination marginal adaptation and surface microstructure of mineral trixide aggregate: a SEM study. *Journal of Dental Research, Dental Clinics, Dental Prospects*.

[37] Asgary S., Parirokh M., Eghbal M. J., Stowe S., Brink F. (2006). A qualitative X-ray analysis of white and grey mineral trioxide aggregate using compositional imaging. *Journal of Materials Science: Materials in Medicine*.

[38] Camilleri J. (2008). The physical properties of accelerated Portland cement for endodontic use. *International Endodontic Journal*.

[39] Oliveira I. R., Andrade T. L., Jacobovitz M., Pandolfelli V. C. (2013). Bioactivity of calcium aluminate endodontic cement. *Journal of Endodontics*.

[40] Wang X., Sun H., Chang J. (2008). Characterization of Ca_3_SiO_5_/CaCl_2_ composite cement for dental application. *Dental Materials*.

[41] PAS (Publicly Available Specification) 132 (2007). *Terminology for the Bio-Nano Interface*.

[42] Gandolfi M. G., Taddei P., Siboni F., Modena E., Ciapetti G., Prati C. (2011). Development of the foremost light-curable calcium-silicate MTA cement as root-end in oral surgery. Chemical-physical properties, bioactivity and biological behavior. *Dental Materials*.

[43] Formosa L. M., Mallia B., Bull T., Camilleri J. (2012). The microstructure and surface morphology of radiopaque tricalcium silicate cement exposed to different curing conditions. *Dental Materials*.

[44] Misra S. K., Ansari T., Mohn D. (2010). Effect of nanoparticulate bioactive glass particles on bioactivity and cytocompatibility of poly(3-hydroxybutyrate) composites. *Journal of the Royal Society Interface*.

[45] Misra S. K., Mohn D., Brunner T. J. (2008). Comparison of nanoscale and microscale bioactive glass on the properties of P(3HB)/bioglass composites. *Biomaterials*.

